# Cases Illustrating the Use of the Pneumatic Aspirator in Surgery

**Published:** 1874-01-15

**Authors:** Charles D. Homans

**Affiliations:** Boston, Mass.


### CASES ILLUSTRATING THE USE OF THE PNEUMATIC ASPIRATOR IN SURGERY.

By Charles D. Homans, M.D., Boston, Mass.

*From the Boston Medical and Surgical Journal.*

THE advantage of the use of the aspirator, in enabling surgeons to make a diagnosis in cases where the existence of fluid is doubtful, seems to be pretty generally recognized; but practitioners do not appear to realize that this instrument is of great value in surgery, in the treatment of many other affections. It has been used for the removal of pus and synovia from joints, for the emptying of chronic abscesses, in cases of chronic hydrocephalus, of retention of urine, of strangulated hernia, and to relieve the pain of distention in cases of great flatulence. In all these cases—some, of necessity, mortal—the relief to pain is very great, while, as a rule, the punctures made by the aspirator needles have been followed by no serious consequences; in fact, in most cases, at *post-mortem* examinations, but little, if any, trace of their passage could be found.

This instrument was used many times, during the past season, in my service at the City Hospital, and the following are some of the most striking of the cases:

CASE I.—*Strangulated Hernia.* April 20th. P. B., laborer, aged 54 years, has had oblique inguinal hernia on the right side for the past ten years; he has always worn a truss, till within a week before entrance; three days ago, after exertion, the hernia came down, and has remained down since, notwithstanding efforts at reduction were made by himself and two physicians. Constitutional disturbance not great. The hernial mass was about the size of a hen's egg, and very tender. The patient was etherized, and taxis tried for half an hour, without success. A fine aspirator needle was then thrust into the tumor, and from three to four drachms of fluid, containing bubbles ef air, drawn out. Taxis was then again resorted to, and the hernia immediately returned. No unfavorable symptoms supervened, and the patient was discharged, well, the eighth day after the operation.

CASE II.—*Strangulated Hernia.* May 26th. B. R., seaman, aged 27 years, entered the hospital with a large inguinal hernia on the right side, which had been down for several hours, and which he had vainly tried to reduce himself. He had been ruptured more than seven years, and had usually worn a truss of his own manufacture. Four years ago, he was operated on by a distinguished surgeon of London, by Wood's method, for the radical cure of the hernia; but the operation, at first apparently successful, was followed by a recurrence of the rupture, after seven or eight months. Since then, it has frequently come down, but he has always been able to return it without the aid of a physician. Now, there is a large hernial tumor in the right groin, very painful and tender. It is quite firm to the touch, and the skin over it shows the scars of the operation in London. There was some acceleration of the pulse, and the countenance was anxious. The patient was etherized, and attempts were made to reduce the hernia by the taxis, by position, and in every way that could be suggested, but without success. The tumor was punctured with the fine needle of the aspirator, three successive times, but no fluid or gas passed out. The ordinary operation for strangulated hernia was then resorted to, and the tumor found to consist wholly of intestine, very tightly compressed, which may, perhaps, explain why no fluid or air came after the punctures. The patient did perfectly well, and was discharged three weeks after the operation.

CASE III. — *Retention of Urine from Stricture.* A man, aged 37 years, entered the hospital with his bladder distended with urine, none having been passed for thirty hours. Many attempts had been made to pass an instrument through the urethra, but without success. There was a stricture four inches from the meatus, and blood followed the attempt to pass the catheter. The fine needle of the pneumatic aspirator was passed into the bladder, behind the pubes, and three pints of urine were drawn off. The bladder was punctured again the next day, after which the urine came naturally.

CASE IV.—A man, aged 28 years, was brought to the hospital, having fallen astride a plank ten hours before, and having been unable to empty his bladder since. He was suffering greatly from distention, and the aspirator was immediately used, as in Case III., forty ounces of urine, slightly tinged with blood, being drawn off. It was afterwards necessary to perform perineal section ; and the man eventually recovered.

Dr. Wm. Ingalls also used the aspirator in a case of retention of urine from stricture, with similar good results ; and it was used many times for emptying abscesses, exploring tumors, etc. Its use in one of the cases of strangulated hernia was, apparently, of the greatest service, while, in the other case, no harm was done, though three punctures were made. In the cases of retention of urine, the advantage of this manner of relieving suffering is certainly very striking, over the old way of tapping through the rectum. The needle is more easily introduced, if a very slight puncture is first made through the skin

